# Transient and Stable Expression of the Neurotensin Receptor NTS1: A Comparison of the Baculovirus-Insect Cell and the T-REx-293 Expression Systems

**DOI:** 10.1371/journal.pone.0063679

**Published:** 2013-05-16

**Authors:** Su Xiao, Jim F. White, Michael J. Betenbaugh, Reinhard Grisshammer, Joseph Shiloach

**Affiliations:** 1 Biotechnology Core Laboratory, National Institute of Diabetes and Digestive and Kidney Diseases, Bethesda, Maryland, United States of America; 2 Membrane Protein Structure and Function Unit, National Institute of Neurological Disorders and Stroke, National Institutes of Health, Rockville, Maryland, United States of America; 3 Department of Chemical and Biomolecular Engineering, Johns Hopkins University, Baltimore, Maryland, United States of America; Cleveland Clinic Lerner Research Institute, United States of America

## Abstract

Nowadays, baculovirus-infected insect cells and tetracycline-inducible mammalian cell lines (T-REx-293) are intensively used for G protein-coupled receptor (GPCR) production for crystallography purposes. Here we constructed a suspension T-REx-293 cell line to stably express an engineered neurotensin receptor 1 (NTS1) mutant and we quantitatively compared this cell line with the transient baculovirus-insect cell system throughout a milligram-scale NTS1 expression and purification process. The two systems were comparable with respect to functional NTS1 expression levels and receptor binding affinity for the agonist [^3^H] neurotensin. However, NTS1 surface display on T-REx-293 cells determined by radio-ligand binding assays was 2.8 fold higher than that on insect cells. This work demonstrates two approaches for preparing milligram quantities of purified NTS1 suitable for structural studies and provides useful input to users in choosing and optimizing an appropriate expression host for other GPCRs.

## Introduction

G protein-coupled receptors (GPCRs) are integral membrane proteins that play a central role in cell signaling by transmitting extracellular signals across membranes to intracellular effector pathways. Closely associated with a wide array of diseases, these proteins are targets for most of the medicines sold worldwide [1]. As of December 2012, structures of 16 unique GPCRs are available (http://blanco.biomol.uci.edu/mpstruc/listAll/list), including the structure of the neurotensin receptor 1 bound to its peptide agonist [2]. The structure determination of GPCRs has been made possible by the supply of ample amounts of correctly folded receptors [3], [4] and progress in purification, crystallization and data collection techniques.

Neurotensin (NT) is a 13 amino acid residue peptide that is found in the nervous system and in peripheral tissues [5]. NT displays a wide range of biological activities and plays important roles in Parkinson’s disease, in pathogenesis of schizophrenia, in modulation of dopamine neurotransmission, hypothermia, antinociception and in promoting the growth of cancer cells [6], [7], [8], [9], [10]. Three neurotensin receptors have been identified. NTS1and NTS2 belong to the class A GPCR family, whereas NTS3 is a member of the sortilin family with a single transmembrane domain [11], [12], [13]. Most of the known effects of NT are mediated through NTS1 [9].

Wild-type rat NTS1 [11] has previously been expressed in *Escherichia coli* (*E. coli*) fused with maltose-binding protein (MBP), and large-scale purification has been accomplished [14]. Recently, systematic scanning mutagenesis of NTS1 has been performed using *E. coli* as the expression host to identify stabilized NTS1 mutants suitable for crystallization [2], [15], [16], [17]. The structure of a stabilized NTS1 mutant (GW5) with T4 lysozyme replacing most of the third intracellular loop, was determined with receptors transiently expressed in the baculovirus-insect cell system [2]. Here we report the expression of NTS1-GW5-Δi3 in a stable, inducible T-REx-293 cell line and in baculovirus-infected insect cells. NTS1-GW5-Δi3 has six stabilizing mutations [2], truncated N- and C-termini, and parts of the third intracellular loop deleted. We refer to this construct in the following as NTS1. We provide a quantitative comparison between the two production hosts regarding aspects of functional NTS1 expression levels and receptor yield after purification, as well as binding properties and cell surface display of the receptors. The scale-up of NTS1 production using T-REx-293 suspension cultures in a bioreactor allows the continued production of the receptor suitable for the application of biophysical analyses such as nuclear magnetic resonance (NMR) spectroscopy and x-ray crystallography.

## Materials and Methods

### Materials

The tritiated agonist [^3^H]NT ([3,11-tyrosyl-3,5-3H(N)]-pyroGlu-Leu-Tyr-Glu-Asn-Lys-Pro-Arg-Arg-Pro-Tyr-Ile-Leu) was purchased from Perkin Elmer. Unlabeled NT was synthesized by the Center for Biologics Evaluation and Research (Food and Drug Administration). The detergents n-decyl-β-D-maltopyranoside (DM), n-dodecyl-β-D-maltopyranoside (DDM), 3-[(3-cholamidopyropyl) dimethylammonio] -1- propanesulfonate (CHAPS) and cholesteryl hemisuccinate Tris salt (CHS) were obtained from Anatrace.

### The NTS1 Construct Used for Expression in Insect Cells and T-REx-293 Cells

The construct NTS1-GW5-Δi3 (here referred to as NTS1) consists of the hemagglutinin signal peptide and the Flag tag [18], [19], followed by the stabilized rat neurotensin receptor NTS1-GW5 (T43-K396 containing the mutations A86L, E166A, G215A, L310A, F358A, V360A) [2] with the intracellular loop 3 residues G275-E296 deleted. A deca-histidine tag was present at the C-terminus. For expression using the baculovirus- insect cell system, NTS1 was subcloned into the transfer vector pFastBac1 (Invitrogen) thus placing NTS1 under the control of the strong polyhedrin promoter. For stable expression in T-REx-293 cells, NTS1 was subcloned into the plasmid pACMV-tetO (a kind gift from Dr. Philip J. Reeves) downstream of the tetracycline-controlled CMV promoter [20].

### Transient Expression of NTS1 in the Baculovirus-insect Cell System

Recombinant baculoviruses were generated using the pFastBac1 transfer plasmid system (Invitrogen). *Trichoplusia ni* cells were infected at a cell density of 0.8–1 million cells/ml with recombinant virus at a multiplicity of infection (MOI) of 5, and the temperature was lowered from 28°C to 21°C (SFX-Insect serum-free medium, HyClone). Cells were harvested by centrifugation 48 hours post infection, resuspended in hypotonic buffer (10 mM Hepes pH 7.5, 10 mM MgCl_2_, 20 mM KCl), flash-frozen in liquid nitrogen and stored at –80°C until use.

### Stable Expression of NTS1 in the T-REx-293 System

The T-REx-293 cell line was maintained as an adherent culture in DMEM containing 10% certified FBS and 5 µg/ml blasticidin (Invitrogen). The cells were transfected with the plasmid pACMV-tetO-NTS1 using Lipofectamine 2000 according to the manufacturer’s protocol (Life Technologies). One day after transfection, cells were transferred into fresh DMEM medium containing 800 µg/ml Geneticin (Cellgro) and the medium was replaced every three days. Two weeks later, fourteen cell clones were separately expanded into two T-flasks each. Cells in one T-flask were harvested during the exponential growth phase and frozen in 10% DMSO for storage. Cells in the other T-flask were induced with 2 µg/ml tetracycline for 24 hrs, after reaching 80% confluency. Cells were then detached from the flask and washed with cold PBS. After adjusting the cell density to around one million cells per ml, protease inhibitors (Roche) were added and the cell suspension was frozen on dry ice in 1 ml aliquots. NTS1 expression levels were determined by [^3^H]NT binding and the clone with the highest expression level was selected for further experiments.

### Adaption of NTS1-expressing T-REx-293 Cells in to Suspension Culture

Three different media were tested for suspension culture of NTS1-expressing T-REx-293 cells: Freestyle™ 293 (Gibco), CD OptiCHO™ (Gibco) and pro293 CD™ Medium (Lonza). The adherent growth medium (DMEM supplemented with 10% certified FBS, 5 µg/ml blasticidin and 500 µg/ml G418) was gradually replaced with the respective suspension growth media during subculture in T-flasks. With the progressive displacement of the adherent growth medium, increasing amounts of viable detached cells were collected, transferred into shake flasks, and maintained in the respective suspension growth media supplemented with 1% certified FBS, 5 µg/ml blasticidin and 500 µg/ml G418. The optimal medium giving highest cell density and viability was chosen for further culture.

### Growth of T-REx-293 Cell Line Suspension Culture in a Bioreactor

The suspension-adapted T-REx-293 cells were grown in 5L of CD OptiCHO medium supplemented with 4 mM L-glutamine, 1% certified FBS, 100 unit/ml penicillin, 100 µg/ml streptomycin and 0.1% pluronic F-68 (Gibco), using a 10L glass bioreactor equipped with a pitch blade impeller connected to a Sartorius BDCU controller. The growth parameters were set to 37°C, pH 7, and 30% dissolved oxygen. The latter two parameters were maintained by interactive control delivery of air and CO_2_ through direct sparge (up to 10 ml/min). The speed of the impeller was 80 rpm. The cell density at inoculation was 3×10^5^ cells per milliliter. At a cell density of 1.5×10^6^ cells per milliliter, expression of NTS1 was induced by addition of 2 µg/ml tetracycline and 2.5 mM sodium butyrate. Cells were harvested 36 hrs after induction, re-suspended in hypotonic buffer (10 mM Hepes pH 7.5, 10 mM MgCl_2_, 20 mM KCl), flash-frozen in liquid nitrogen and stored at –80°C until use.

### Analytical Solubilization of NTS1

Cell pellets from 10 ml of suspension cultures were suspended in Tris-glycerol-NaCl buffer. Then the detergent DM and CHS were added to give a final buffer composition of 50 mM TrisHCl pH 7.4, 200 mM NaCl, 30%(v/v) glycerol, 1% (w/v) DM, and 0.1%(w/v) CHS in a total volume of 2.5 ml. The samples were placed on a rotating mixer at 4°C for 1 hour. Cell debris and non-solubilized material were removed by ultracentrifugation (TL100 rotor, 60 k rpm, 4°C, 30 min in Optima Max bench-top ultracentrifuge, Beckman), and the supernatants containing detergent-solubilized NTS1 were used to determine the total number of expressed receptors by a detergent-based radio-ligand binding assay (see below).

### Purification of NTS1 Produced in T-REx-293 and Insect Cells

All buffer volumes relate to 1L of original cell culture. T-REx-293 cells were thawed and the volume was brought to approximately 200 ml with hypotonic buffer. The cells were then re-suspended using a Turrax T-25 (IKA) homogenizer at 8,200 rpm for 2 min. After centrifugation (45 Ti rotor, 40,000 rpm, 45 min, 4°C, Optima L90K, Beckman), the membranes were resuspended (Turrax T-25) in approximately 120 ml of high-salt buffer (10 mM Hepes pH 7.5, 1 M NaCl, 10 mM MgCl_2_, 20 mM KCl) supplemented with DNaseI (final concentration 10 µg/ml) and AEBSF (100 µM), and centrifuged again. The high-salt washes were repeated 4 more times with the DNaseI addition omitted after the 2^nd^ wash. All subsequent steps were performed at 4°C or on ice, and AEBSF (100 µM final concentration) was repeatedly added throughout the procedure. The washed membranes were resuspended in a final volume of 40 ml of buffer (62.5 mM TrisHCl pH 7.4, 625 mM NaCl, 37.5% (v/v) glycerol) containing 10 µM neurotensin peptide. NTS1 was extracted by drop-wise addition of 10 ml of a 5%(w/v) DM/0.5%(w/v) CHS solution. After 2.5 hours, the sample was clarified by centrifugation (45 Ti rotor, 40,000 rpm, 1 hour, Optima L90K, Beckman), adjusted with imidazole to a final concentration of 20 mM, and then passed through a 0.2 µm filter (Stericup). Next, the sample was loaded at a flow rate of 0.2 ml/min onto 2 ml Talon resin packed into an XK16 column (GE Healthcare) equilibrated with Talon-A buffer (50 mM TrisHCl pH 7.4, 30% (v/v) glycerol, 500 mM NaCl, 20 mM imidazole, 0.1%(w/v) DM/0.01%(w/v) CHS) containing 1 µM neurotensin peptide. After washing with 29 column volumes of buffer Talon-A, NTS1 was eluted with Talon-B buffer (50 mM TrisHCl pH 7.4, 30%(v/v) glycerol, 500 mM NaCl, 200 mM imidazole, 0.1%(w/v) DM/0.01%(w/v) CHS) containing 5 µM neurotensin peptide. Peak fractions were combined (5 ml total volume) and analyzed. The purification of NTS1 from insect cells was performed in a similar manner.

### Protein Analysis and Radio-ligand Binding Assays

The protein content was measured according to the Amido Black method of Schaffner and Weissmann [21] with bovine serum albumin as the standard. Western blot analysis was performed as described [22] using the INDIA HisProbe-HRP reagent (Pierce) and the substrates 3,3′-diaminobenzidine tetrahydrochloride and H_2_O_2_.

[^3^H]NT ligand-binding assays with intact cells were carried out in 500 µl of TEBB assay buffer (50 mM Tris-HCl pH 7.4, 1 mM EDTA, 0.1% bovine serum albumin, 40 µg/ml bacitracin) containing 10 nM [^3^H]NT and about 100,000 cells. After incubation for 2–4 hours on ice, separation of bound from free ligand was achieved by rapid filtration through GF/B glass fiber filters (Whatman) pretreated with polyethylenimine. The amount of radioactivity was quantified by liquid scintillation counting (Beckman LS6500). Non-specific [^3^H]NT binding of 4160 dpm was subtracted from total binding to calculate the receptor density at the cell surface. The concentration of [^3^H]NT used in these assays was four-fold above the apparent dissociation constant for membrane-bound NTS1 [2] to allow high receptor occupancy, but it kept nonspecific [^3^H]NT binding to a minimum.

**Figure 1:**
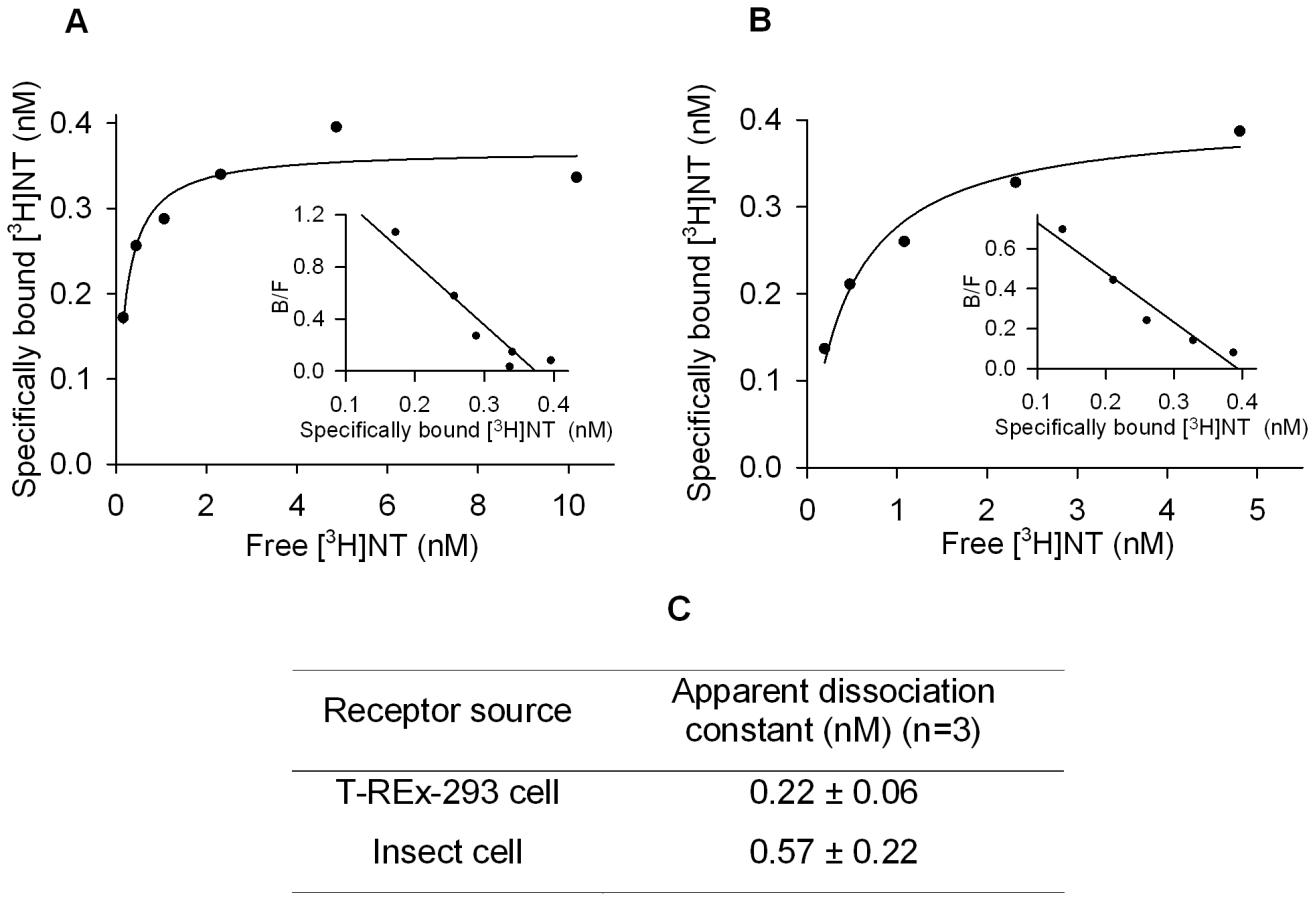
[^3^H]NT saturation binding of NTS1 expressed in (A) T-REx-293 cells and (B) insect cells. NTS1 was extracted from membranes with the detergent DM/CHS and subjected to radio-ligand binding analysis. Inset: Scatchard transformation. Representative experiments conducted as single data points are shown. (C) Table summarizing the values of the apparent dissociation constants values for [^3^H]NT binding. These values are not statistically different (P = 0.2, unpaired two-tailed t-test). Data are collected from three repeated experiments.

Ligand-binding assays with detergent-solubilized receptors were carried out in TEBB assay buffer containing 0.1% (w/v) DDM, 0.2% (w/v) CHAPS, 0.04% (w/v) CHS. For one-point assays, receptors were incubated with 2 nM [^3^H] NT on ice for 1 hour in a volume of 150 µl. The concentration of [^3^H] NT used here was at least 3.5-fold above the apparent dissociation constants for detergent-solubilized NTS1 (Fig. 1) to allow high receptor occupancy. Separation of the receptor-ligand complex from free ligand (100 µl) was achieved by centrifugation-assisted gel filtration using Bio-Spin 30 Tris columns (BioRad), equilibrated with RDB buffer (50 mM Tris-HCl, pH 7.4, 1 mM EDTA, 0.1%(w/v) DDM, 0.2%(w/v) CHAPS, 0.04%(w/v) CHS). Non-specific [^3^H]NT binding of 220 dpm was subtracted from total binding to calculate the total amount of receptors in HEK-293 and insect cells. The number of functional NTS1 was estimated by specific [^3^H]NT binding assuming one ligand-binding site per receptor molecule. The number of cells in the assay was derived by cell counting at cell harvest. This approach led to the calculation of the parameter “receptors/cell”.

For saturation binding experiments, the [^3^H]NT concentration was varied from 0.2 nM to 10 nM. Non-specific [^3^H]NT binding was determined in the presence of 50 µM unlabeled NT. Data were analyzed by nonlinear regression using GraphPad Prism software (version 4, GraphPad Software) and best fit to a one-site binding equation to determine the apparent dissociation constants for NTS1 produced in insect and HEK-293 cells. Note that the saturation binding experiments using the NTS1 mutant did not reach equilibrium within the incubation time because of the very slow agonist off-rates determined in a previous study [2]. Individual experiments were conducted as single data points.

## Results

### NTS1 Expression in Suspension T-REx 293 Cells and Insect Cells

**Figure 2:**
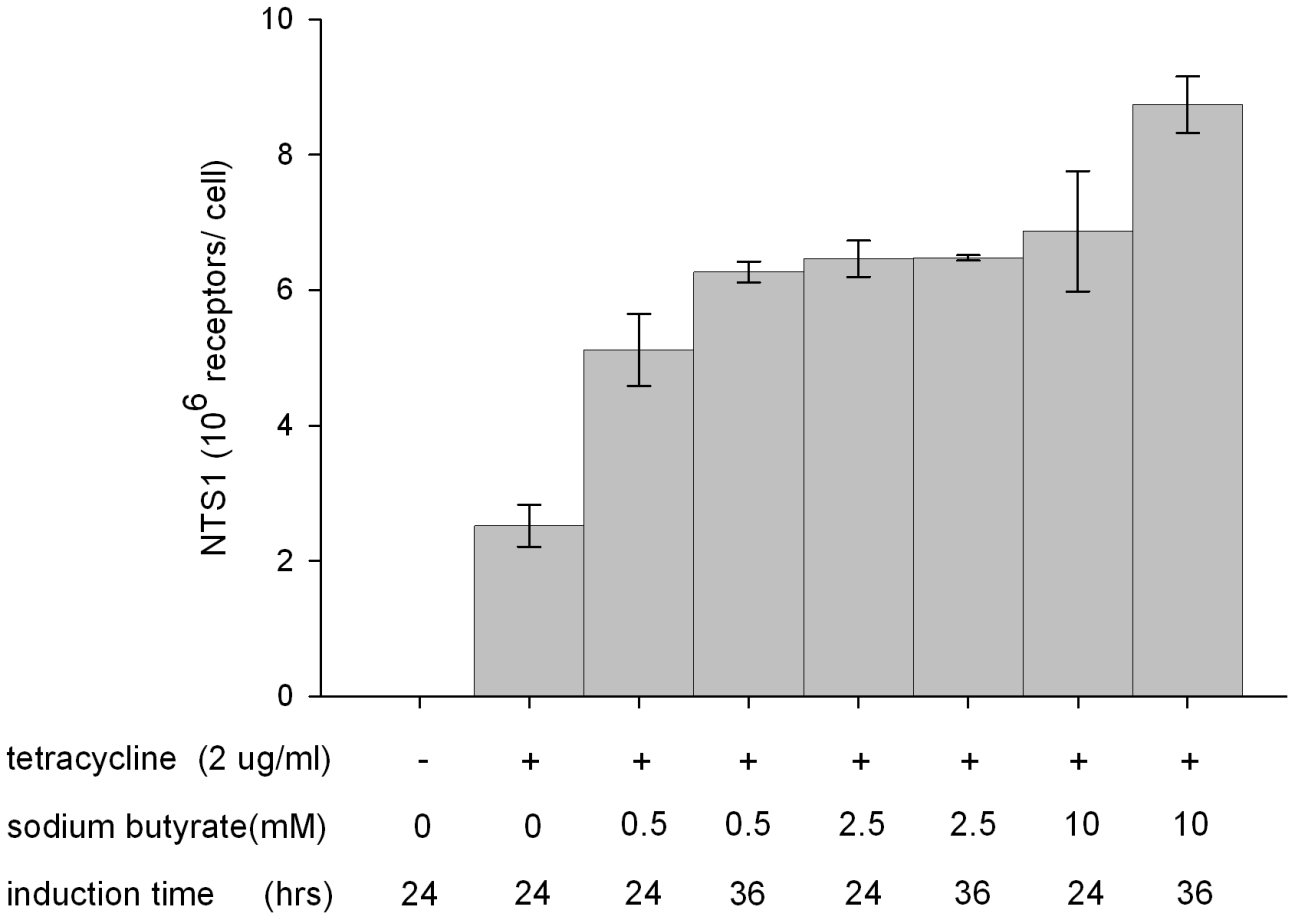
Optimization of NTS1 expression under different induction conditions using a stable T-REx-293 cell line. The data are collected from a selected high-expressing clone. Cells were grown in suspension in CD OptiCHO medium supplemented with 4 mM L-glutamine and 1% certified FBS and were induced in the late exponential growth phase (at a viable cell density of 2 million cells/ml) with tetracycline. The addition of sodium butyrate enhanced expression levels. Intact cells were subjected to [^3^H]NT binding assay to determine the number of receptors located at the cell-surface. For all conditions, n = 2, error bars indicate SEM (standard error of the mean).

The N-terminally truncated rat NTS1 with 6 stabilizing mutations was stably expressed in the tetracycline-regulated T-REx-293 cell line. A high-expressing clone was selected and adapted step-wise to suspension culture. The CD OptiCHO™ medium was selected for scaling-up because it supported highest cell density and viability among the three different media tested. In CD OptiCHO™ supplemented with 4 mM L-glutamine and 1% certified FBS, the clone grew to a density of 4 million cells/ml in shake flasks with viability higher than 95% and a doubling time of 48 hours. To maximize the production of NTS1, a preliminary orthogonal array design [23] was carried out investigating three induction parameters: tetracycline concentration (1–4 µg/ml), sodium butyrate (NaBu) concentration (0.5–10 mM) and induction time (24–60 hrs). Initial variance analysis showed a negligible impact by higher tetracycline doses, a significant effect of NaBu, and considerable cell death with induction times longer than 48 hrs (data not shown). Therefore, further optimization efforts were focused on the NaBu dose with induction times of 24 or 36 hrs. As shown in Fig. 2, NTS1 expression was undetectable in the absence of tetracycline, while in the presence of 2 µg/ml tetracycline, 2.5 million plasma membrane localized receptors were produced. The expression of functional NTS1 improved with increasing NaBu concentrations (0.5–10 mM) and optimal production was achieved by the addition of 2 µg/ml tetracycline and 10 mM NaBu when the viable cell density reached 2 million cells/ml, with harvest 36 hrs later. These optimized conditions resulted in 8.8 million copies of plasma membrane localized NTS1 (Fig. 2), a 3.5-fold increase of cell surface expression compared to induction with tetracycline alone.

Pilot-scale production of NTS1 was carried out next in a 5L bioreactor. Two µg/ml tetracycline and 2.5 mM NaBu were added to the T-REx-293 cells when the viable cell density reached 1.5 million cells/ml. Cells were harvested 36 hrs after induction and plasma membrane localized receptors were determined to be 5.7 million per cell with [^3^H]NT binding assays on intact cells. To determine the total amount of functional NTS1 (i.e. receptors residing in membranes of the endoplasmic reticulum and the Golgi apparatus, and in the plasma membrane), cells were solubilized with detergent and the number of receptors was determined by a detergent-based radio-ligand binding assay. This resulted in 12.7 million receptors per cell and a yield of 1.0 mg of functional NTS1 per liter culture (Table 1).

**Table 1:** Purification of NTS1 from different host cells.

Host cell	Cell density at harvest (10^6^cells/ml)	Functional receptor copies (10^6^/cell)	Functional receptor yield (mg/L)	Talon eluate (mg/L)
Insect cells	1.6^a^	12.3^a^	1.3^a^	1.5
T-REx-293 cells	1.2^b^	12.7^b^	1.0^b^	0.9

Average data for insect cells are from four purification experiments using 1L (3 experiments) or 4L (1 experiment) of cell culture as starting material. Average data for T-REx-293 cells are from two purification experiments using 1L of culture as starting material. All purification procedures were performed in the presence of neurotensin. The functional receptor yield was calculated from the cell density at harvest and [^3^H]NT binding assays on detergent-solubilized cells. The protein yield of the Talon column eluate was determined by the Amido Black method, as the presence of NT during the purification procedure prevented a radio-ligand binding analysis. Because of contaminants in the Talon column eluate, the content of NTS1 is overestimated.

a average data from seven 5L cultures,

b data from one 5L bioreactor run. The calculated molecular mass of NTS1 (without signal peptide) is 39560 Da.

Both Sf9 and *Trichoplusia ni* cells were tested as insect hosts for NTS1 expression, exploring temperature and time as variables. We used cell viability as the read-out, as only healthy cells have an intact machinery for insertion and folding of membrane proteins. We observed reduced cell viability after infection when cells were kept at 27°C. In contrast, cell viability was higher when the temperature was lowered to 21°C after infection. The best results were obtained using *T. ni* cells at 48 hrs post-infection (cell viability >94%). All subsequent pilot-scale expression experiments were therefore performed using *T. ni* cells, with reduction of the temperature to 21°C after infection, and harvest at 48 hrs post-infection. Based on the average of 7 independent expression experiments, NTS1 was produced at a total number of 12.3 million receptors per cell or 1.3 mg receptor per liter culture.

### Purification of NTS1 from T-REx-293 Cells and Insect Cells

**Figure 3:**
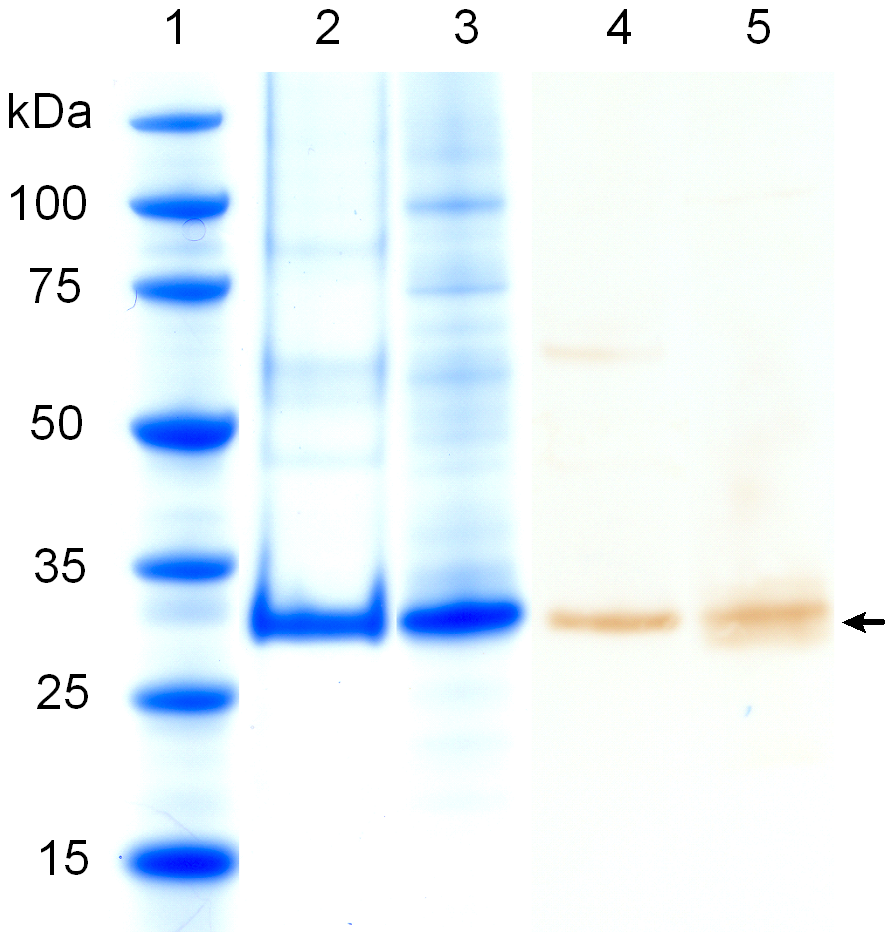
Purification of NTS1. The progress of purification was monitored by SDS-PAGE (NuPAGE 4–12% Bis-Tris gel, Invitrogen, 1× MES SDS buffer) and SimplyBlue staining. The arrow indicate the NTS1 band. Lane 1: Novagen Perfect Protein Marker (15–150 kDa); lane 2: Talon eluate of NTS1 produced in T-REx-293 cells (3.5 µg); lane 3: Talon eluate of NTS1 produced in insect cells (6 µg); Western blot analysis of total cell extract was performed using the HisProbe-HRP reagent recognizing the histidine tag. Lane 4: NTS1 produced in T-REx-293 cells (122,000 lyzed cells with 113 ng functional NTS1); lane 5: NTS1 produced in insect cells (110,500 lyzed cells with 107 ng functional NTS1).

The presence of neurotensin enhances the stability of NTS1 [2]; therefore, all purification steps were conducted in the presence of the agonist peptide. The purification of NTS1 produced in T-Rex-293 and insect cells was done in one step by immobilized metal affinity chromatography (Talon resin) in the presence of the detergent DM/CHS. The yield of NTS1 per liter of cell culture, as calculated from the cell density at harvest and [^3^H]NT binding assays using detergent-solubilized cells, was similar for both expression hosts (Table 1). The protein content of the Talon column eluates was determined by the Amido Black method, as the presence of NT during the purification procedure prevented a radio-ligand binding analysis. Note that the Amido Black method overestimated the content of purified NTS1 because of contaminants present in the Talon column eluate (Fig. 3). Of interest, it appears that the amount of minor contaminants in the Talon column eluate from T-Rex-293 material is less than that from insect cells (compare lanes 2 and 3 in Fig. 3), possibly indicating the presence of different histidine-rich proteins in both expression hosts.

### Characterization of NTS1 Produced in Insect Cells and T-Rex-293 Cells

**Figure 4:**
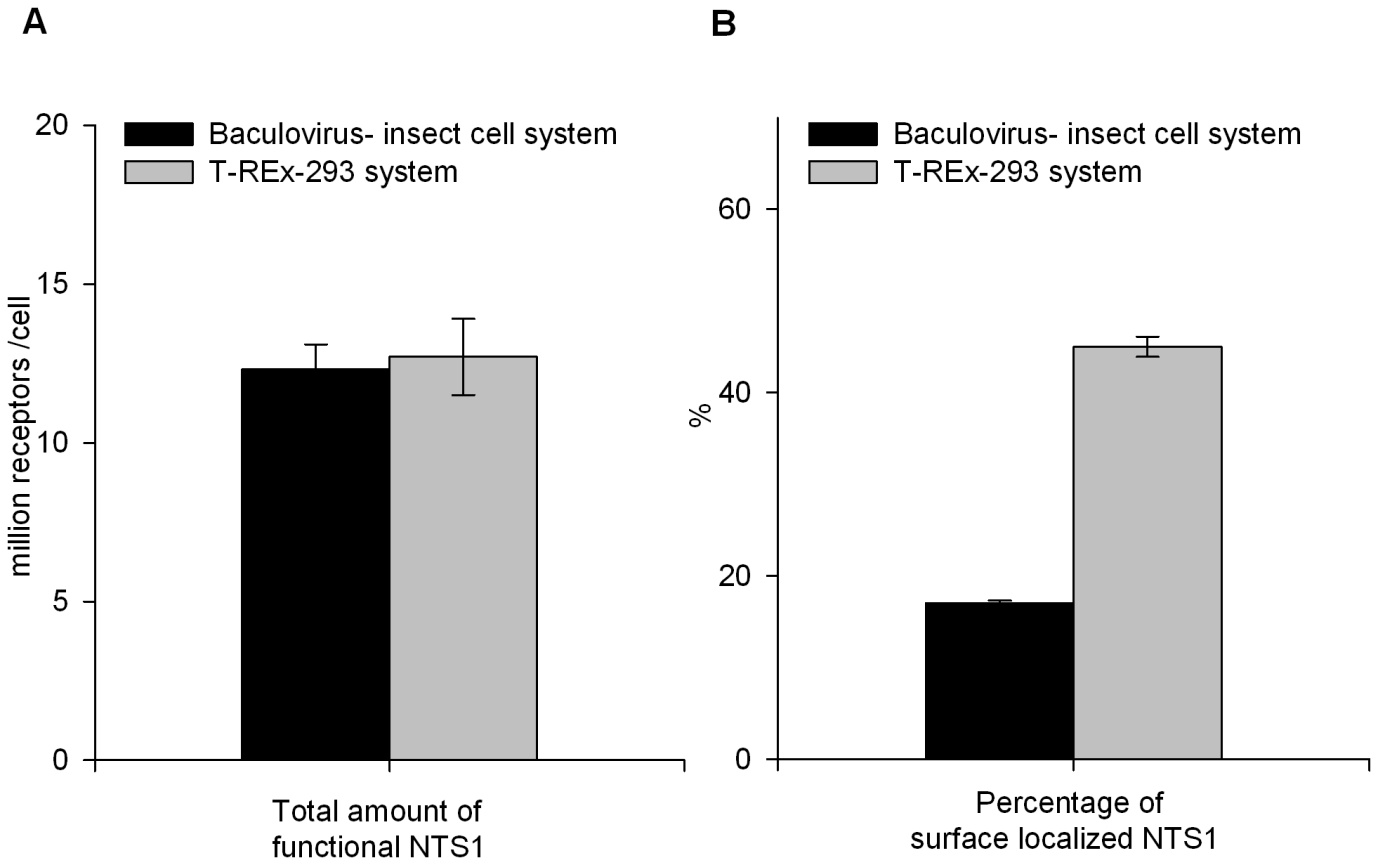
Expression of NTS1 in the transient insect cell system and inducible T-REx-293 system. (A) Total functional NTS1 numbers were determined by [^3^H]NT binding assays using detergent solubilized cells (B) Surface localized NTS1 numbers were determined by [^3^H]NT binding assays using intact cells and combined with data from (A) to calculate percentage of surface localized NTS1 (baculovirus insect cell system: 7 independent expression experiments; T-REx-293 system: 4 independent measurements on one 5L expression experiment). The expression of NTS1 in insect cells was conducted as described in Materials and Methods. Expression of NTS1 in the T-REx-293 system was induced by the addition of 2 µg/ml tetracycline and 10 mM sodium butyrate, with harvest and analysis 36 hours later.

To quantify the total amount of NTS1 and the amount of plasma membrane localized NTS1, [^3^H]NT binding assays on detergent-solubilized cell extracts and on intact cells were conducted. The total number of functional NTS1 per cell, produced in T-Rex-293 cells and insect cells, was similar (Fig. 4). T-Rex-293 cells produced 12.7 million receptors per cell, while insect cells produced 12.3 million receptors per cell. However, the percentage of NTS1 molecules that had trafficked to the cell surface was 2.8-fold higher in the case of T-Rex-293 cells compared to insect cells (Fig. 4).

To estimate the degree of NTS1 misfolding in both expression hosts, we conducted Western blot analysis of solubilized protein from T-Rex-293 and insect cells, using the INDIA HisProbe reagent which detects both functional and misfolded histidine tagged NTS1. Equivalent amounts of correctly folded NTS1 (as determined by [^3^H]NT binding assays) from the two hosts were subjected to gel electrophoresis and subsequent Western blot analysis. Comparable band intensities indicated the expression of similar amounts of total, i.e. correctly folded and misfolded, NTS1, thus suggesting a similar propensity for receptor misfolding in T-Rex-293 and insect cells (Fig. 3, lanes 4 and 5).

The ligand binding property of the receptors produced in the two hosts were comparable according to saturation binding assays using detergent-solubilized receptors. The apparent dissociation constants for NTS1 from both hosts were not statistically different (Fig. 1).

## Discussion

Unveiling the structures of GPCRs by crystallography will help to elucidate the mechanism of many diseases and enhance potential drug discovery and development. In order to accomplish this, milligram amounts of functional receptors are needed. Seeking an appropriate expression host is vital, as the host can affect the quantity and quality of the starting material used for purification and crystallization [24]. Possible host choices include bacteria [25], yeast [26], the baculovirus-insect cell system [19], and mammalian cells [27]. The baculovirus-insect cell system and the inducible T-REx-293 system were intensively investigated in recent years because of their lipid composition, translocation machinery and protein folding capabilities [28]. In this study, NTS1 served as a model GPCR and the baculovirus-insect cell and mammalian T-REx- 293 systems were compared throughout NTS1 expression and purification process in a quantitative way.

By using an inducible expression strategy, 2.5 million functional copies of NTS1 per cell were detected in the plasma membrane of T-Rex-293 cells (Fig. 2); this level of expression is 167-fold higher than that of NTS1 expressed constitutively in HEK-293T cells [29]. Optimization of the induction parameters led to an increase in production to 8.8 million functional receptors per cell. Among the three induction parameters tested (tetracycline concentration, NaBu concentration and duration of induction), the concentration of NaBu had the greatest impact. This compound has successfully been applied together with tetracycline for the expression of many GPCRs [30], [31]. NaBu is routinely used at low concentration (1–5 mM [32]), possibly because of its cytotoxic effects on cell growth at higher concentrations [33]. In the experiments reported here, NaBu was used at concentrations as high as 10 mM resulting in improved NTS1 expression (Fig. 2). The enhancement of NTS1 gene transcription by inhibition of histone deacetylase may be a possible explanation for this observation. It is also possible that NaBu led to growth arrest of the host cells, allowing metabolic energy be channeled towards NTS1 production [34].

Expression levels and binding properties of functional NTS1 from the T-REx-293 and baculovirus-insect cell systems were comparable. For both systems, the yields were approximately 12 million functional receptors per cell or about 1 milligram per liter culture after purification. Hence both expression hosts are suitable for generating NTS1 in quantities sufficient for structural studies. From a production point of view, a significant difference was observed in the surface-presentation of the receptors. In T-REx-293 cells, 2.8 times more NTS1 trafficked to the plasma membrane compared to insect cells, as determined by radio-ligand binding assays. In the case of the serotonin transporter, higher surface-presentation was correlated with an increased population of correctly folded transporters [24]. However, when equal amounts of functional NTS1 from both hosts were analyzed by Western blot using the INDIA HisProbe reagent capturing the C-terminal histidine tags of both functional and non-functional NTS1, similar staining intensities were observed. This indicated comparable NTS1 folding efficiencies in both host systems. The fact that the structure of NTS1 was determined from material produced transiently in insect cells [2] suggests that the differences between surface-located and internal receptors are at best subtle. More studies will be needed in order to understand the implications of difference in receptor surface- presentation.

**Figure 5:**
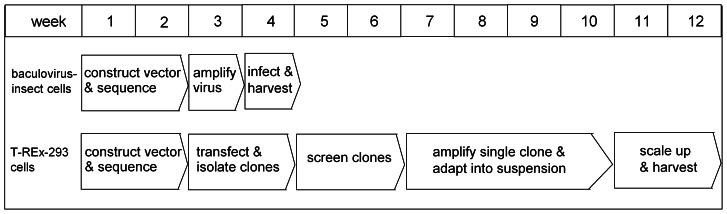
Timeframe for the establishment of the transient baculovirus-insect cells system and stable expression with inducible T-REx-293 system for GPCR expression.

The timeframe required for process development and for establishing the expression conditions for the transient insect cell system and the stable T-Rex-293 cell system are different (Fig. 5). The estimated time needed for large-scale expression in baculovirus-infected insect cells is around 6 weeks, starting from the transfer plasmid. In contrast, it can take up to 12 weeks to construct the T-REx-293 cell line stably expressing NTS1. Based on this time frame, the transient baculovirus-insect cell expression system is better suited at an early stage of structural work when the GPCR of interest is subject to frequent sequence modifications for construct optimization. Transient transfection of HEK-293 cells may be considered an alternative to the baculovirus system, bypassing the step for generating recombinant baculoviruses [35]. Stable expression in suspension T-REx-293 cells becomes preferable for the production of GPCRs of a specified construct due to its ease of handling. The well-established scale-up methods for suspension cultures in a bioreactor allow for the one-step large-scale production of sufficient amounts of receptors for applications of biophysical techniques such as NMR spectroscopy. Cost-wise, the prices for media for T-REx-293 and insect cells are comparable. It is likely possible that improvement in high-density suspension culture of T-REx-293 cells will further reduce the cost per milligram of protein.

In conclusion, we generated a stable suspension-adapted T-REx-293 cell line capable of expressing 1 milligram functional NTS1 per liter culture. This cell line was found to be comparable to the transient baculovirus-insect cell system in regard to functional NTS1 expression level and receptor binding properties. The ease of handling, processing in a bioreactor, and not requiring continued virus amplification make the T-REx-293 cell line ideally suited for long-term production of NTS1.
